# Long-read sequencing transforms the diagnosis of congenital adrenal hyperplasia: resolving pseudogene interference and structural variations

**DOI:** 10.3389/fped.2025.1603819

**Published:** 2025-06-27

**Authors:** Junfeng Zeng, Xiaoling Huang, Yanwei Li, Tizhen Yan, Jiwu Lou, Guizhen Lyu, Yanjin Li

**Affiliations:** ^1^ Newborn Screening Center, Dongguan Maternal and Children Health Hospital, Dongguan, Guangdong, China; ^2^ Dongguan Key Laboratory of Screening, Diagnosis and Treatment of Neonatal Genetic and Metabolic Diseases, Dongguan, Guangdong, China; ^3^ Dongguan Key Laboratory of Clinical Medical Test Diagnostic Technology for Oncology, Dongguan Labway Clinical Laboratory Co., Ltd., Dongguan, Guangdong, China; ^4^ Prenatal Diagnostic Center, Dongguan Maternal and Children Health Hospital, Dongguan, Guangdong, China; ^5^ Dongguan Molecular Diagnostic Technology and Infectious Disease Medical Test Engineering Research Center, Dongguan Labway Clinical Laboratory Co., Ltd., Dongguan, Guangdong, China

**Keywords:** congenital adrenal hyperplasia, long-read sequencing, *CYP21A2*, *HSD3B2*, pseudogene interference

## Abstract

**Background:**

Congenital adrenal hyperplasia (CAH) is an autosomal recessive disorder primarily caused by defects in adrenal steroidogenesis. Conventional genetic methods struggle to resolve complex structural variations and pseudogene interference in key genes like *CYP21A2*. Our study will evaluate the efficacy of Long-Read Sequencing (LRS) as a comprehensive diagnostic tool for CAH, demonstrating its ability to simultaneously detect large structural variations, single nucleotide variants (SNVs), and small insertions or deletions.

**Methods:**

Four probands with clinically diagnosed CAH underwent detailed biochemical profiling, including serum 17-hydroxyprogesterone, serum sodium and serum potassium. Genomic DNA was extracted from peripheral blood and subjected to LRS using Single-Molecule Real-Time (SMRT) Technologies (Pacifc Biosciences). A targeted panel covering the *CYP21A2* and *HSD3B2* genes, as well as other genes related to CAH was captured. Bioinformatic analysis included alignment with Minimap2, variant calling with Sniffles2 and Medaka, and phasing analysis to resolve pseudogene interference.

**Results:**

LRS identified compound heterozygous and homozygous variants in *CYP21A2* (e.g., c.293-13C > G, c.518T > A, CH-1) and novel compound heterozygous variants in *HSD3B2* (c.121G > T and c.757T > G). In combination with biochemical tests, clinical manifestations, and the ACMG guidelines, these gene mutations were the cause of the patient's disease. LRS resolved pseudogene interference and provided unambiguous cis/trans phasing.

**Conclusion:**

LRS is a robust diagnostic tool for CAH, offering comprehensive detection of genetic variants, including large deletions and SNVs in both *cis* and *trans* forms. Its ability to resolve pseudogenes and structural variations positions LRS as a first-tier diagnostic tool for CAH, improving accuracy, streamlining clinical workflows and ultimately benefits patients.

## 1 Introduction

Next-generation sequencing (NGS), or high-throughput sequencing, has revolutionized the clinical diagnosis of genetic diseases by providing high-throughput, accurate, and cost-effective sequencing of large portions of the genome (1). Techniques such as targeted (panel) sequencing and whole exome sequencing (WES) have emerged as key methods for genetic diagnostics, significantly improving detection rates, which now range from 20% to 50% for many genetic disorders (2, 3). Despite these advancements, NGS technologies face challenges in accurately detecting mutations in genes with highly homologous sequences, particularly in regions containing pseudogenes. These problematic regions, first identified in 1977 using cloning techniques (4), are dispersed throughout the human genome, with around 11,000 pseudogenes identified to date (5). As pseudogenes share significant sequence homology with functional genes, they can cause misinterpretations in genetic testing, leading to false-positive or false-negative results (6). The short read length typical of NGS platforms exacerbates this issue, making it difficult to differentiate between real and pseudogene sequences, especially in genes that are crucial for diagnosing specific disorders. One such disorder is CAH, a group of autosomal recessive genetic conditions characterized by defects in the adrenal cortical steroid synthesis pathway (7). CAH primarily manifests as impaired corticosteroid synthesis and androgen overproduction, leading to a variety of clinical symptoms. Among the different types of CAH, 21-hydroxylase deficiency (21-OHD) is the most prevalent, accounting for over 95% of all cases (8). Traditional genetic diagnostic methods, including multiplex ligation-dependent probe amplification (MLPA) and locus-specific PCR followed by Sanger sequencing, are commonly used but have significant limitations (9). These methods are prone to interference from pseudogenes due to the high sequence similarity between the *CYP21A2* gene, which is responsible for 21-OHD, and its pseudogene, *CYP21A1P*. The presence of these pseudogenes can result in the exchange of gene sequences, complicating the interpretation of results and potentially leading to misdiagnoses. In contrast, LRS also known as third-generation sequencing (TGS), offers several advantages over conventional NGS methods. LRS provides long read lengths, allowing for full-length sequencing of DNA and the ability to map entire regions without the need for PCR amplification. This capability enables more accurate detection of complex variants, including large deletions and structural variations, which are often missed by traditional sequencing methods. LRS has proven effective in resolving challenging genomic regions, such as those containing pseudogenes or highly homologous sequences, making it an ideal tool for diagnosing genetic diseases like CAH. These advancements in sequencing technology offer promise for improving diagnostic accuracy and facilitating more reliable genetic testing for complex conditions like CAH.

This study demonstrates LRS's superiority in diagnosing four CAH probands with diverse variants, including novel *HSD3B2* mutations. We validated LRS as a robust method for resolving pseudogenes, detecting SV, and phasing alleles, thereby addressing critical gaps in current diagnostic workflows, as evidenced by its ability to significantly improve SV detection and allele phasing accuracy over traditional short-read sequencing technologies.

## 2 Subjects and methods

### 2.1 Study design

**Figure 1:**
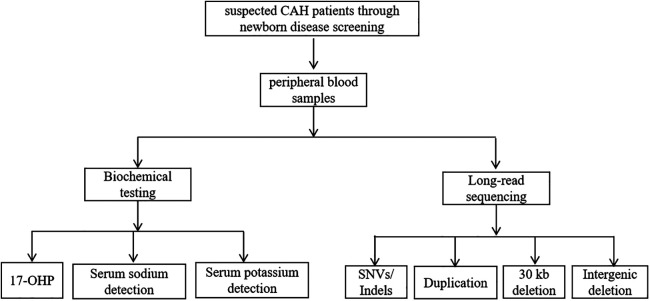
Study design flow chart. Flowchart showing suspected CAH patients screened through newborn disease screening, leading to peripheral blood samples. The samples undergo biochemical testing and long-read sequencing. Biochemical testing includes 17-OHP, serum sodium detection, and serum potassium detection.

Our study recruited patients suspected of having CAH through newborn disease screening. Peripheral blood samples were collected from these patients and subsequently subjected to both biochemical testing and LRS for genetic analysis (Figure 1). The biochemical tests included measurement of 17-OHP levels (normal level is less than 11.5 nmol/L), serum potassium detection (normal level is 3.5–5.3 mmol/L), and serum sodium detection (normal level is 135–150 mmol/L), which were used to assess adrenal function and electrolyte balance. Additionally, LRS was performed to detect SNVs/Indels, Duplication, 30 kb deletion and Intergenic deletion in the target genes. By integrating molecular diagnostics with clinical manifestations, physical examination, and biochemical tests, a definitive diagnosis of CAH was made, followed by standardized treatment.

### 2.2 Clinical data

Proband 1 was a 3-year and 4-month-old boy from Family 1, born after a normal pregnancy via caesarean section at 39 weeks of gestation (2021-02-04). He weighed 4.25 kg (+2SD), measured 51 cm in length (<+1SD), and had a head circumference of 34 cm at birth. The scrotum was darkly pigmented, but no external genital malformations, such as hypospadias, were observed. His parents were healthy, with no family history of endocrine disorders. His mother, aged 25, had a spontaneous abortion 2 years prior. Postnatally, he was artificially fed, demonstrated a good appetite, and exhibited no issues with sucking, swallowing, frequent vomiting, or abnormal stool or urine patterns. On the third day after birth (2021-02-07), neonatal screening revealed an abnormally high 17-OHP level of 80.41 nmol/L, prompting clinical suspicion of CAH.

Proband 2 was a 4-month-old girl, born after a normal pregnancy (first parity) at 39+ weeks of gestation through natural delivery (2024-05-02). She weighed 3.25 kg (−1SD to +1SD) and measured 50 cm in length (−1SD to +1SD) at birth. Artificial feeding was initiated postnatally, and she has since exhibited a good appetite, normal behaviour, and no frequent vomiting. On the third day of life (2024-05-05), neonatal screening revealed an elevated 17-OHP level of 80.41 nmol/L, prompting clinical suspicion of CAH.

Proband 3 was a 1-month-old boy, the product of a third gestation and first parity, born naturally at 40+ weeks of gestation (2024-08-12). He weighed 3.35 kg (<+1SD), measured 50 cm (−1SD to +1SD) in length, and had a head circumference of 33 cm. He had no history of asphyxia or resuscitation. Postnatally, he was mixed-fed but presented with poor appetite and lethargy, requiring hospitalization in the neonatal department due to hypoglycaemia and jaundice. He was discharged after 1 week of care, consuming 60 ml of milk per feeding. On the fourth day after birth (2024-08-16), neonatal screening revealed a significantly elevated 17-OHP level of 646.4 nmol/L, prompting clinical suspicion of CAH.

Proband 4 was a 3-month-old girl born after a normal pregnancy (first parity) at 37 weeks of gestation via natural delivery (2024-05-26). At birth, her amniotic fluid was clear, and there was no history of asphyxia or resuscitation. Her weight was 2.77 kg (−2SD to −1SD) and her length was 48.5 cm (−1SD to +1SD). Her parents were healthy, with no family history of genetic disorders. After birth, she was fed with a combination of breast milk and formula, had a good appetite, and occasionally experienced milk regurgitation. Newborn screening revealed an elevated 17-OHP level of 205.1 nmol/L (2024-05-29), prompting clinical suspicion of CAH.

### 2.3 Long-read sequencing

Doctors will provide parents with health education through a short video before they make an informed decision about genetic testing. Blood samples from the probands and their parents were collected to extract genomic DNA. Long-fragment multiplex PCR amplification targeted regions encompassing CAH-associated genes (*CYP21A2*, *CYP11B1*, *CYP17A1*, *HSD3B2*, *STAR*, *CYP11A1*, and *POR*). The amplified fragments were ligated with sequencing universal adapters to form SMRT dumbbell libraries. These libraries were further combined with sequencing primers and enzymes to create sequencing libraries. The libraries were hybridized into sequencing chips for processing with a gene sequencer. The sequencing data were analysed using bioinformatics software to detect and annotate variants (10).

### 2.4 Protein structure and analysis

For the prediction analysis of the effects of gene missense mutation on protein secondary structure, stability, and electrostatic potential, please refer to our previously published article (11).

## 3 Results

### 3.1 Proband 1

Proband 1 was the son of a Chinese couple who had a spontaneous abortion 2 years ago.

He was initially recalled for evaluation on day 16 (2021-02-20). Serum potassium (5.82 mmol/L), and serum sodium (136.4 mmol/L), cytogenetic analysis, testicular and adrenal ultrasounds, were normal. But, the 17-OHP level (142.1 nmol/L) was still high. Hormonal assays revealed that testosterone was low at 72.59 ng/dl (normal range: 128–813 ng/dl), while other indicators were normal: serum dehydroepiandrosterone sulphate was 15.7 μg/dl (normal range: 3.4–124 μg/dl), androstenedione was 1.31 nmol/L (normal range: 0.21–2.72 nmol/L), and cortisol was 94.98 nmol/L (normal range: 68.2–327 nmol/L).

#### 3.1.1 Treatment and follow-up

Hydrocortisone was administered orally at a dose of 4 mg/d, divided into three doses. During intermittent follow-up evaluations, he experienced hyponatremia (129.9 mmol/L) at 1.5 months of age. Oral sodium supplementation was initiated at 1 g/d, which helped maintain serum sodium levels within a low-normal range of 130–133 mmol/L. At 5 months of age, serum sodium levels dropped to 126 mmol/L, and 17-OHP levels rose to 349.95 nmol/L. Fludrocortisone was added at a dose of 0.05 mg/d. Regular monitoring of electrolytes and hormones was continued, with subsequent results showing normal electrolyte levels, including serum sodium ranging from 138 to 145 mmol/L and serum potassium from 3.8 to 5.3 mmol/L. 17-OHP levels fluctuated between 26 and 50 nmol/L, although it could reach as high as 78–90 nmol/L during periods of missed medication due to illness or before follow-up visits. Scrotal pigmentation normalized by 6 months of age.

#### 3.1.2 Growth and development

Weight and height were within normal limits until the age of 2 years, with a height of 75 cm at 1 year and 89 cm at 2 years. Growth velocity slowed after 2 years of age. At 3 years old, the height was 93 cm (−1SD), and weight was 12 kg (−2SD < • < −1SD). Hydrocortisone dosage remained at 4 mg/d, with a calculated body surface area dose of 7.7 mg/(m^2^·d). At 3 years and 3 months old, height was 93.5 cm (−2SD < • < −1SD), 17-OHP was abnormally low at 10 nmol/L, serum cortisol was 104.2 ng/ml, and bone age was 3.8 years. The treatment regimen was adjusted: hydrocortisone tablets were reduced to 3.33 mg/d and fludrocortisone was decreased to 0.025 mg/d. At 3 years and 6 months, height was 96 cm (−2SD < • < −1SD), weight was 13 kg (−2SD < • < −1SD), 17-OHP level was risen (34.7 nmol/L).

#### 3.1.3 Genetic findings

**Figure 2:**
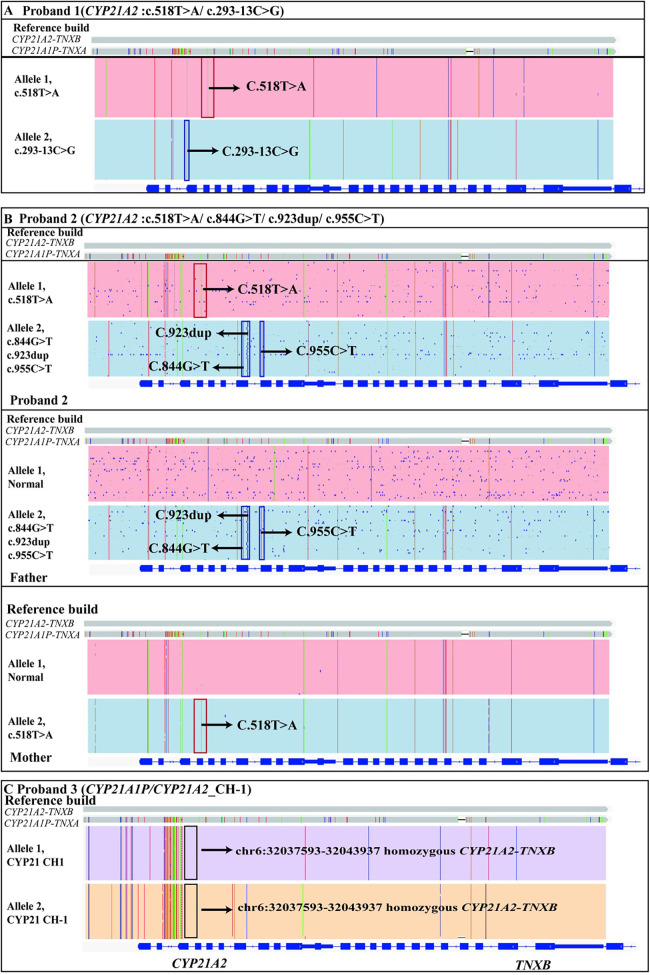
The genetic results of proband 1, proband 2 and proband 3 identified by LRS. **(A)** The proband 1 had two variants c.518T > A(p.I173N) and c.293-13C > G in the *CYP21A2* gene, which were identified by LRS and found to be in trans. The red box above indicated the c.518T > A mutation in the *CYP21A2* gene and the blue box below indicated the c.293-13C > G mutation. **(B)** The proband 2 had four variants in the *CYP21A2* gene identified by LRS. Of these, c.844G > T(p.V282l), c.923dup(p.L308Ffs*6) and c.955C > T(p.Q319*) were inherited from her father, and c.518T > A(p.I173N) was inherited from her mother. **(C)** The proband 3 had a homozygous variant in the pseudo-fusion gene *CYP21A2-TNXB* at the location chr6:32037593-32043937, which was also identified by LRS. A chimeric *CYP21A1P/CYP21A2* genes mutation (chimera CH-1) had occurred. Arrows indicate specific mutations.

LRS detected two heterozygous variants in the *CYP21A2* gene: a simple masculinizing variant c.518T > A and a salt-losing variant c.293-13C > G. Both variants were found to be in trans (Figure 2A; Table 1).

**Table 1:** Clinical phenotype and genetic test results of 4 probands.

Patient	Gender	Date of birth	Clinical phenotype	Gene	hg38 location	Variant	In trans	Inheritance	Population frequency (gnomAD)	Evidence	Pathogenicity	Benefits of LRS
Proband 1	Male	2021/2/4	Dark scrotum, elevated 17-OHP, high potassium, low sodium	*CYP21A2 (NM_000500)*	chr6:32039426	c.518T > A (p.I173N)	Allele 1	-	0.00157687	PM3_VeryStrong + PS3 + PP1+PM2_supporting	Pathogenic	Adjusting the dosage of medication, maintaining normal electrolyte levels, and incorporating lifestyle guidance led to catch-up growth in height, approaching that of normal children.
					chr6:32039081	c.293-13C > G	Allele 2	-	0.00271739	PM3_VeryStrong + PS3 + PP1-Strong + PM2_supporting	Pathogenic	
Proband 2	Female	2024/5/2	Elevated 17-OHP, high potassium, low sodium	*CYP21A2 (NM_000500)*	chr6:32039426	c.518T > A (p.I173N)	Allele 1	Maternal	0.00157687	PM3_VeryStrong + PS3 + PP1+PM2_supporting	Pathogenic	Clarifying the etiology increased treatment adherence, and currently, growth and development, as well as motor and language development, are normal.
					chr6:32040110	c.844G > T (p.V282l)	Allele 2	Paternal	0.0276147	PM3_VeryStrong + PS3 + PS1+PM2_supporting	Pathogenic	
					chr6:32040182	c.923dup (p.L308Ffs*6)			0.00019305	PVS1 + PM3_Strong + PM2_supporting		
					chr6:32040421	c.955C > T (p.Q319*)			0.0128205	PVS1 + PS3 + PM2_supporting		
Proband 3	Male	2024/8/12	Dark penis and scrotum, elevated 17-OHP, high potassium, low sodium	*CYP21A2 (NM_000500)*	NA	CH-1	Allele 1 + 2	-	NA		Pathogenic	Clarifying the etiology increased treatment adherence, and currently, growth and development, as well as motor and language development, are normal.
Proband 4	Female	2024/5/26	Macroclitoridis, elevated 17-OHP, high potassium, low sodium	*HSD3B2(NM_000198)*	chr1:119415540	c.121G > T (p.E41*)	Allele 1	-	NA	PVS1 + PM2_supporting	Likely pathogenic	Clarifying the etiology increased treatment adherence, and currently, growth and development, as well as motor and language development, are normal.
					chr1:119422258	c.757T > G (p.Y253D)	Allele 2	-	NA	PM3 + PP3 + PM2_supporting	Versus uncertain significance	

Homozygous mutation (c.293-13C > G) at this locus had been reported in multiple patients. Additionally, pathogenic or likely pathogenic variants had been detected in trans with the variant of interest in multiple patients (12–16) (PM3_VeryStrong). Functional studies had suggested that the variant leaded to impaired gene function (17) (PS3). The variant had been reported to cosegregate with the disease in multiple affected family members (16, 18, 19) (PP1_Strong). The variant was absent in the ChinaMAP database. Its frequencies in the 1000 Genomes Project database, the Exome Aggregation Consortium (ExAC) database, and the Genome Aggregation Genome Aggregation Database (gnomAD) were 0.000998403, 0.00235660191731302, and 0.00271739, respectively (PM2_supporting). Integrating the clinical phenotype, biochemical test results, and the ACMG guidelines (20), the gene mutation (c.293-13C > G) was defined as a pathogenic mutation (PM3_VeryStrong + PS3 + PP1-Strong + PM2_supporting) (Table 1).

Homozygous missense mutation c.518T > A (p.I173N) had been detected in multiple CAH patients (13, 21). Pathogenic or likely pathogenic variants have been reported in trans with the variant of interest in multiple CAH patients (12, 22, 23) (PM3_VeryStrong). The variant was absent in the ChinaMAP database. Its frequencies in the ExAC database, the 1000 Genomes Project database, and the gnomAD database were 0.000403670447385003, 0.000798722, and 0.00157687, respectively (PM2_supporting). The variant had been reported to cosegregate with the disease in two affected family members (one instance of infectious cosegregation) (18) (PP1). Functional studies had suggested that the variant leaded to impaired gene function (24) (PS3). Integrating the clinical phenotype, biochemical test results, and the ACMG guidelines (20), the mutation (c.518T > A) was defined as a pathogenic mutation (PM3_VeryStrong + PS3 + PP1 + PM2_supporting) (Table 1).

### 3.2 Proband 2

Proband 2 was the daughter of a Chinese couple. Newborn screening revealed elevated 17-OHP levels. On re-examination at 8 days of age, the 17-OHP level was risen (25.8 nmol/L). At 1 month of age, the 17-OHP level increased to 87.3 nmol/L, and adrenal ultrasound showed no abnormalities.

#### 3.2.1 Treatment and follow-up

At the follow-up visit at 1.5 months of age, laboratory tests revealed elevated serum potassium (6.18 mmol/L) and low serum sodium (133.6 mmol/L). Hormonal assays showed 17-OHP at 86.2 nmol/L, testosterone at 22.77 ng/dl, cortisol at 48.2 ng/ml, androstenedione at 4.260 ng/ml, and serum dehydroepiandrosterone sulphate at 63.6 μg/dl.

Treatment was initiated with hydrocortisone at a dose of 2.5 mg/d [current weight 4.1 kg, calculated dose 10.26 mg/(m^2^·d)], administered orally in three divided doses. Fludrocortisone was given at a dose of 0.05 mg/d, taken orally in two doses. At 1.9 and 2.1 months of age, 17-OHP levels were slightly reduced, but hyponatremia and hyperkalaemia persisted. Consequently, the fludrocortisone dose was increased to 0.066 mg/d. At 2.5 months of age, 17-OHP levels were lower than previously recorded, and electrolyte levels normalized. At 4 months of age, laboratory tests showed 17-OHP level at 15.2 nmol/L, testosterone at 18.21 ng/dl, cortisol at 38.1 ng/ml, androstenedione at 2.10 ng/ml, and serum dehydroepiandrosterone sulphate at 55.6 μg/dl.

#### 3.2.2 Genetic findings

We identified a heterozygous maternal missense mutation c.518T > A (p.I173N) in *CYP21A2* gene. Its evidence for the pathogenic classification of this variant had been analyzed in Proband 1. Additionally, we also identified three heterozygous paternal variants (c.844G > T, c.923dup, and c.955C > T) in *CYP21A2* gene (Figure 2B).

The homozygous missense mutation c.844G > T (p.V282l) had been reported in multiple Chinese patients, and pathogenic variants had been detected in trans with the variant of interest in multiple patients (25–27) (PM3_VeryStrong). Functional studies had suggested that the variant leaded to impaired gene function (24, 28, 29) (PS3). The variant resulted in the same amino acid change as the known pathogenic variant c.844G > C (PS1). The variant was absent in the ChinaMAP database and the 1000 Genomes Project database. Its frequencies in the ExAC and the gnomAD databases were 0.0104249370016224 and 0.0276147, respectively (PM2_supporting). Integrating the clinical phenotype, biochemical test results, and the ACMG guidelines (20), the mutation (c.844G > T) was defined as a pathogenic mutation (PM3_VeryStrong + PS3 + PS1 + PM2_supporting) (Table 1).

The frameshift mutation [c.923dup (p.L308Ffs*6)] resulted in a change in the gene open reading frame, thereby altering the protein function (PVS1). In two reported patients, pathogenic variants were detected in trans with the variant of interest (30) (PM3_Strong). The variant was absent in the ChinaMAP database, the ExAC database, and the 1000 Genomes Project database, and its frequency in the gnomAD database was 0.00019305 (PM2_supporting). Integrating the clinical phenotype, biochemical test results, and the ACMG guidelines (20), the mutation ((c.923dup) was defined as a pathogenic mutation (PVS1 + PM3_Strong + PM2_supporting) (Table 1).

The nonsense mutation c.955C > T (p.Q319*) resulted in a premature stop codon, thereby altering the protein function (PVS1). Functional studies had suggested that the variant leaded to impaired gene function (19) (PS3). The variant was absent in the ChinaMAP database, the ExAC database, and the 1000 Genomes Project database, and its frequency in the gnomAD database was 0.0128205 (PM2_supporting). Integrating the clinical phenotype, biochemical test results, and the ACMG guidelines (20), the mutation ((c.923dup) was defined as a pathogenic mutation (PVS1 + PS3 + PM2_supporting) (Table 1).

### 3.3 Proband 3

Proband 3 was the son of a Chinese couple. Neonatal screening indicated elevated 17-OHP levels, which remained high at 652.9 nmol/L when retested 10 days after birth. Physical examination revealed darkened penis and scrotum. Biochemical analysis showed serum potassium at 5.85 mmol/L and serum sodium at 122.9 mmol/L. Hormonal assays revealed testosterone at 680.70 ng/dl, serum dehydroepiandrosterone sulphate >1,000 μg/dl, androstenedione at 12.11 nmol/L, and cortisol at 9.35 nmol/L.

#### 3.3.1 Treatment and follow-up

The treatment regimen included fludrocortisone at 0.05 mg per dose, administered twice daily, hydrocortisone at 5 mg/d divided into three oral doses, and 10% sodium chloride at 10 ml/d divided into three oral doses. After initiating this treatment and conducting regular monitoring, 17-OHP levels gradually decreased, and electrolyte levels normalized. At 1 month of age, follow-up hormonal assays showed androstenedione at 3.4 nmol/L, testosterone at 165 ng/dl, dehydroepiandrosterone sulphate at 114 μg/dl, and cortisol at 120 nmol/L.

#### 3.3.2 Genetic findings

Genetic analysis identified a known homozygous pathogenic variant in chimeric *CYP21A1P*/*CYP21A2* genes (chimera CH-1) associated with the salt-wasting form of congenital adrenal hyperplasia in Proband 3 (31) (Figure 2C; Table 1). The pathogenic mechanism of this structural variant is as follows: during the pachytene stage of meiosis, unequal crossing over occurs in a part of the RP-C4-CYP21-TNX (RCCX) modules between non-sister chromatids, leading to the formation of a fusion gene between the *CYP21A2* gene and the pseudogene *CYP21A1P*. To date, nine types of fusion genes have been reported internationally, namely chimera CH-1 to CH-9 (31).

### 3.4 Proband 4

Proband 4 was the daughter of a Chinese couple. At 16 days of age, she was recalled for a physical examination, during which stable vital signs were noted along with an enlarged clitoris. Re-examination revealed persistently elevated 17-OHP level at 215.6 nmol/L. Laboratory tests showed serum potassium at 5.82 mmol/L, serum sodium at 131.5 mmol/L, testosterone at 320.70 ng/dl, serum dehydroepiandrosterone sulphate >1,000.00 μg/dl, androstenedione at 10.64 nmol/L, and cortisol at 7.65 nmol/L. At 20 days of age, re-examination of electrolytes showed serum potassium at 5.85 mmol/L and serum sodium at 129.8 mmol/L.

#### 3.4.1 Treatment and follow-up

Treatment was initiated with hydrocortisone at 3 mg/d, divided into three oral doses (based on a dosage of 15 mg/m^2^), fludrocortisone at 0.1 mg/d, divided into two oral doses, and 10% sodium chloride solution at 10 ml/d, divided into five doses. Following treatment, electrolyte levels gradually normalized, and 17-OHP levels decreased but remained above the normal level.

At 1.5 months of age, the dose of hydrocortisone was increased to 3.25 mg/d (based on a body weight of 4 kg and a body surface area of 0.24 m^2^, corresponding to 13.5 mg/m^2^; divided into three doses of 1.25, 1, and 1 mg), while fludrocortisone and sodium supplementation remained unchanged. Subsequent monitoring showed a gradual decrease in 17-OHP levels and a reduction in clitoral size. Electrolyte levels continued to normalize, and 17-OHP level further decreased.

At 2 months of age, hormonal assays showed androstenedione at 2.8 nmol/L, testosterone at 180 ng/dl, dehydroepiandrosterone sulphate at 126 μg/dl, and cortisol at 106 nmol/L. At 3 months of age, androstenedione was 1 nmol/L, testosterone was 79 ng/dl, dehydroepiandrosterone sulphate was 100 μg/dl, and cortisol was 88 nmol/L.

#### 3.4.2 Genetic findings

**Figure 3:**
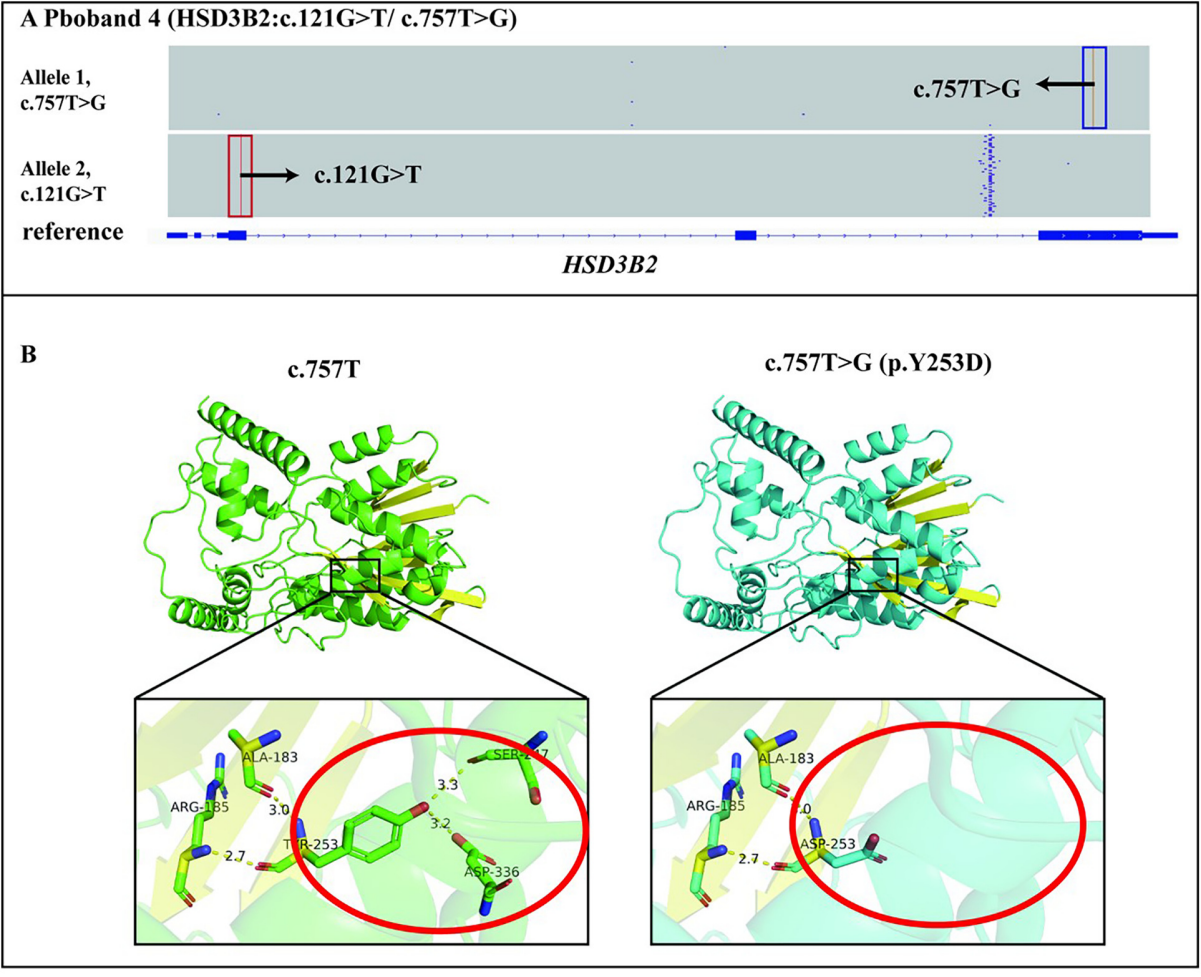
Genetic diagnosis of the proband 4 and the effect of the c.757T > G(p.Y253D) variant on tertiary structure. **(A)** The proband 4 had two variants c.757T > G(p.Y253D) and c.121G > T(p.E41*) in the *HSD3B2* gene, which were identified by LRS were in trans. **(B)** The c.757T > G(p.Y253D) variant affected the main-chain structure of *HSD3B2*.

We identified two trans heterozygous variants: a nonsense variant c.121G > T (p.E41*) and a missense variant c.757T > G (p.Y253D) in *HSD3B2* gene (Figure 3A). The variant c.121G > T (p.E41*) resulted in a premature stop codon, thereby altering the protein function (PVS1). This variant was not found in the human genome mutation frequency databases mentioned earlier (PM2_supporting). Integrating the clinical phenotype, biochemical test results, and the ACMG guidelines (20), the mutation c.757T > G (p.Y253D) in *HSD3B2* gene was defined as a likely pathogenic mutation (PVS1 + PM2_supporting) (Table 1).

The c.757T > G (p.Y253D) variant was analysed using the Consurf conservation analysis tool. The wild-type amino acid at position 253 is relatively conserved in terms of burial, with a conservation score of 8 (scoring range 1–9), suggesting that the variant at position 253 may disrupt the protein's burial, potentially leading to decreased stability and interfering with specific interactions between the protein and its ligands, substrates, or other biological molecules. Further analysis using Alphafold3 modelling and PyMOL mapping revealed that the c.757T > G (p.Y253D) variant disrupted the distance between the main-chain ASP253 and SER247 and ASP336 residues, which formed hydrogen bonds after the variant. This indicated that the variant affected the main-chain structure of HSD3B2 (Figure 3B). Taken together, these findings suggested that the variant impacted the tertiary structure of HSD3B2 (PP3). Proband 4 was found to have a likely pathogenic variant (c.121G > T) in trans with the variant of interest (c.757T > G) in the *HSD3B2* gene (PM3). This variant was not found in the human genome mutation frequency databases mentioned earlier (PM2_supporting). Integrating the clinical phenotype, biochemical test results, and the ACMG guidelines (20), the missense mutation c.757T > G (p.Y253D) in *HSD3B2* gene was defined as a l vs. uncertain significance mutation (PM3 + PP3 + PM2_supporting) (Table 1).

## 4 Discussion

In this study, four CAH probands were analysed, revealing *CYP21A2* gene variants in three probands and compound heterozygous variations in the *HSD3B2* gene—NM_000198:c.121G > T (p.E41*) and c.757T > G (p.Y253D)—in one proband (Table 1). The variants identified in the three CAH probands with *CYP21A2* mutations included compound heterozygous variants NM_000500:c.293-13C > G and c.518T > A (p.I173N), c.518T > A (p.I173N) and c.844G > T (p.V282l), c.923dup (p.L308Ffs*6), c.955C > T (p.Q319*), and homozygous deletions of CYP21 CH-1. These findings highlighted the genetic complexity of CAH and the role of *CYP21A2* as a major contributor to disease aetiology.

CAH is a rare genetic disorder with a prevalence of approximately 1:10000 to 1:20000, varying by ethnicity, geographical region, and diagnostic practices. Non-classic CAH, however, occurs more frequently, with prevalence estimates ranging from 1:200 to 1:2000 (8, 10). Classic CAH, particularly when caused by 21-hydroxylase (21OH) deficiency, can lead to life-threatening adrenal crises in neonates if left untreated. Standard management involves lifelong glucocorticoid replacement therapy, such as hydrocortisone, and mineralocorticoid replacement therapy, like fludrocortisone, to correct salt-wasting symptoms in neonatal and early infancy stages (32–34). Timely initiation of glucocorticoids following newborn screening for elevated 17-OHP levels is critical for improving survival and quality of life. In this study, after newborn screening revealed elevated levels of 17-OHP, we promptly initiated treatment and follow-up monitoring with hydrocortisone. As a result, there was a significant improvement in their quality of life (Table 1). Genetic testing remains the gold standard for diagnosing CAH, particularly in cases with ambiguous clinical or biochemical findings or in patients already on glucocorticoid therapy. Genetic analysis facilitates confirmation of diagnosis and informs management strategies (35). After the genetic testing clarified the etiology for our patients, we were able to precisely adjust the dosage of medications. Moreover, adherence to the treatment regimen increased. The parents strictly followed the regular follow-up appointments and the lifestyle guidance. As a result, the children exhibited catch-up growth, with their heights approaching that of normal children (Table 1).

Key genes implicated in CAH include *CYP21A2* (OMIM 613815), *CYP11B1* (OMIM 610613), *CYP17A1* (OMIM 609300), *HSD3B2* (OMIM 613890), *STAR* (OMIM 600617), *CYP11A1* (OMIM 118485), and *POR* (OMIM 124015). Current molecular detection approaches such as NGS, MLPA, and Sanger sequencing are widely employed. However, NGS struggles to differentiate *CYP21A2* from its highly homologous pseudogene, *CYP21A1P*, while MLPA and Sanger sequencing are better suited for this task.

In clinical practice, testing typically begins with MLPA and Sanger sequencing for *CYP21A2*, proceeding to NGS for other genes if results are negative. This sequential strategy reflects the predominance of *CYP21A2*-related 21OH deficiency, which accounts for over 95% of CAH cases (8). However, this approach may prolong the diagnostic cycle, increase costs, and erode patient confidence, particularly if results are inconclusive. Furthermore, the reliance on 17-OHP levels for newborn screening is associated with high false-positive rates, particularly in preterm or low-birth-weight infants, and approximately 30% of CAH cases may remain undetected (36–40). LRS has emerged as a powerful alternative for the diagnosis of genetic disorders, including CAH, Fragile X syndrome, thalassemia, and spinal muscular atrophy. LRS offers advantages such as long read lengths, high single-base accuracy, and rapid detection, allowing it to detect complex structural variations and distinguish homologous regions with high GC content (41, 42). Unlike NGS, LRS can comprehensively identify both known and novel variants and determine whether variants are in cis or trans configurations without requiring parental genetic analysis. Specifically for CAH, LRS can accurately classify six common deletions (*CYP21 CH-3*, *CH-5*, *CH-8* and *TNX-CH1*, *CH2*, *CH3*) that are challenging to detect using MLPA or Sanger sequencing (43).

Despite its benefits, LRS has limitations, including a higher sequencing error rate and cost compared to conventional methods. However, its ability to simultaneously analyse all seven CAH-related genes reduces the need for sequential testing, offering a more cost-effective and comprehensive diagnostic solution. The integration of LRS in CAH newborn screening could improve diagnostic accuracy and reduce false positives, paving the way for precise genotyping and effective management of this disorder. Future research should focus on optimizing LRS technology to address its limitations and facilitate its adoption in routine clinical practice. Future research should focus on addressing these limitations to enhance the utility of LRS in clinical diagnostics. Efforts should prioritize reducing costs through technological advancements and increased scalability, which could enable broader adoption of LRS in routine genetic testing. The development of error-correction algorithms and hybrid diagnostic approaches combining LRS with NGS could further improve accuracy and reliability. Furthermore, integrating LRS into newborn screening programs could revolutionize CAH diagnosis by providing comprehensive genotyping from a single test, reducing diagnostic delays, and minimizing false positives. Large-scale studies evaluating the cost-effectiveness and clinical outcomes of LRS-based screening protocols are necessary to establish their feasibility for widespread implementation.

## 5 Conclusion

This is the first report of the *HSD3B2* variants c.121G > T (p.E41*) and c.757T > G (p.Y253D), which expanded the variant spectrum of *HSD3B2*. LRS is a robust diagnostic tool for CAH, offering comprehensive detection of genetic variants, including large deletions and SNVs in both cis and trans forms. Its ability to distinguish pseudogenes and structural variants makes LRS a first-tier CAH diagnostic tool. This improves accuracy, simplifies clinical workflows, and ultimately benefits patients. Patients then actively cooperate with treatment, greatly improving their quality of life.

## Data Availability

The original contributions presented in the study are included in the article/Supplementary Material. Further inquiries can be directed to the corresponding authors.
